# Mechanism of Action of Mesenchymal Stem Cell-Derived Exosomes in the Intervertebral Disc Degeneration Treatment and Bone Repair and Regeneration

**DOI:** 10.3389/fcell.2021.833840

**Published:** 2022-01-14

**Authors:** Weishi Liang, Bo Han, Yong Hai, Duan Sun, Peng Yin

**Affiliations:** Department of Orthopedic Surgery, Beijing Chaoyang Hospital, Capital Medical University, Beijing, China

**Keywords:** exosomes, mesenchymal stem cells (MeSH ID D059630), intervertebral disc degeneration (IDD), bone repair and regeneration, mechanism of action

## Abstract

Exosomes are extracellular vesicles formed by various donor cells that regulate gene expression and cellular function in recipient cells. Exosomes derived from mesenchymal stem cells (MSC-Exos) perform the regulatory function of stem cells by transporting proteins, nucleic acids, and lipids. Intervertebral disc degeneration (IDD) is one of the main causes of low back pain, and it is characterized by a decreased number of nucleus pulposus cells, extracellular matrix decomposition, aging of the annulus fibrosus, and cartilage endplate calcification. Besides, nutrient transport and structural repair of intervertebral discs depend on bone and cartilage and are closely related to the state of the bone. Trauma, disease and aging can all cause bone injury. However, there is a lack of effective drugs against IDD and bone injury. Recent MSC-Exos fine tuning has led to significant progress in the IDD treatment and bone repair and regeneration. In this review, we looked at the uniqueness of MSC-Exos, and the potential treatment mechanisms of MSC-Exos with respect to IDD, bone defects and injuries.

## 1 Introduction

**FIGURE 1:**
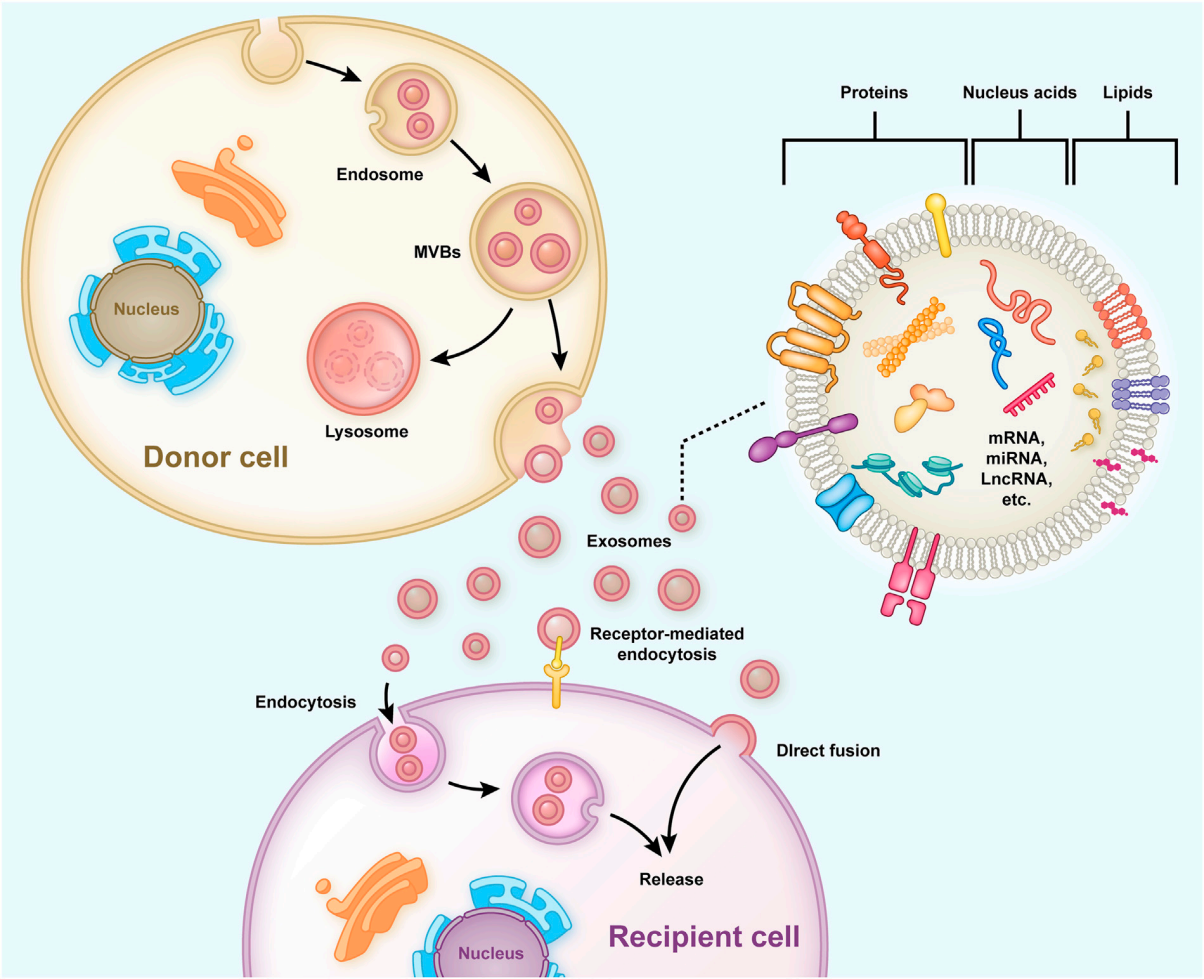
Typical process of exosome generation, secretion, and transfer from the donor cells to the recipient cells, and the exosome structure is shown. Early endosomes containing ILVs are formed preliminarily and then developed into mature MVBs to release and form exosomes into the extracellular. The exosomes have a bilayered membrane structure containing functional proteins, nucleic acids (mRNA, miRNA, lncRNA, etc.), and lipids, some of which are released into recipient cells to regulate gene expression and cell function.

Exosomes are bilayered extracellular functional vesicles released by different cells with a diameter ranging between 40–120 nm (Simons and Raposo, 2009). In the early stages, endosomes containing intraluminal vesicles (ILVs) are formed preliminarily, and then large mature multivesicular bodies (MVBs) within the cell release ILVs into the extracellular space to form exosomes (Zhang et al., 2019) (Figure 1). Exosomes carry out their functions by fusing with cell membranes or binding membrane proteins of the recipient cells. They contain functional proteins, nucleic acids (mRNA, miRNA, lncRNA, etc.) and lipids, and are carriers of intercellular communication between donor and recipient cells (Wang et al., 2021b). MiRNA is a crucial communication medium contained in exosomes, which can regulate the expression of genes and proteins in recipient cells and inhibit the degradation of exosomes (Valadi et al., 2007; Jong et al., 2012). After entering target cells, exosomal miRNA binds to target gene mRNA through partial sequence complementation and participate in tissue repair, inflammation, apoptosis and other processes, thus playing an important role in the regulation of gene expression (Ti et al., 2016; Chen et al., 2019). Exosomes originate from a wide range of sources, and almost all cells can secrete exosomes. The exosomes secreted under normal and pathological conditions are different, even for the same cells (Zhang et al., 2015). If exosomes fail to bind to their target cells in time, they are rapidly metabolized. When applied topically or injected throughout the body, they can provide multiple therapeutic benefits, such as repairing the damaged intervertebral discs and bone tissues (Riau et al., 2019).

MSCs with multidirectional differentiation and self-replication potential have long been considered as an effective method for repairing intervertebral disc disorders and bone injuries, but there are many safety problems (Uccelli et al., 2008; Guadix et al., 2017). Many studies have demonstrated that exosomes derived from mesenchymal stem cells (MSC-Exos) have similar biological effects to MSCs in terms of tissue regeneration and repair functions (Cosenza et al., 2017). The application of MSCs avoids the problems associated with intact cell transplantation, such as immune rejection, infection, and non-directed cell differentiation (Lou et al., 2017). Exosomes secreted by MSCs from bone marrow, adipose, umbilical cord can all promote tissue regeneration and repair (Zhou et al., 2019; Li et al., 2020a; Zhang et al., 2021c). MSCs of different ages can regulate each other through exosomes, while younger MSC-Exos can enhance the proliferation and osteogenic differentiation of older MSCs (Jia et al., 2020).

IDD is associated with various factors such as aging, abnormal biomechanical burden, reduced nutrient supply to the cartilage endplates (Dowdell et al., 2017). In IDD, the water content of the nucleus pulposus decreases, and the pressure load decreases (Adams and Roughley, 2006). At the same time, the annulus fibrosus carries more load and is, therefore, more prone to damage. The healing potential of intervertebral discs without vascular nourishment is low, and there is no effective treatment to inhibit or even repair IDD (Urban and Roberts, 2003). Besides, the health of the surrounding bone and cartilage is closely related to the overall condition of the intervertebral disc, since the disc receives its nutrients from the endplate’s blood supply (Geer, 2018). Although new materials can reduce bone defects, it is still necessary to explore bioactive substances that can promote bone regeneration and repair (Dimitriou et al., 2011; Amini et al., 2012). Therefore, we focused on acellular MSC-Exos over the past years to elucidate their potential effectiveness on IDD treatment and bone regeneration and repair. In this review, we looked at the relationships between MSC-Exos and IDD, MSC-Exos and bone repair and regeneration, and further discussed the mechanism of action of MSC-Exos on the treatment of IDD and promoting bone repair and regeneration.

## 2 Mesenchymal Stem CELL-DERIVED Exosomes and Intervertebral Disc Degeneration

### 2.1 Relationship Between MSC-Exos and Intervertebral Disc Degeneration

**FIGURE 2:**
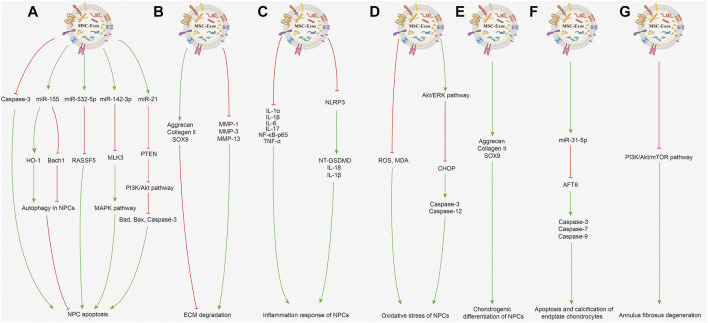
Potential mechanisms of MSC-Exos for treating IDD. **(A)** Inhibiting NPC apoptosis and promoting NPC proliferation. MSC-Exos alleviate NPC apoptosis induced by acidic pH through repressing caspase-3 expression and attenuating caspase-3 cleavage. BMSC-Exos can increase miR-155 expression to upregulate HO-1 expression and downregulate Bach1 expression, subsequently activating autophagy in NPCs to inhibit the cell apoptosis in the state of low blood supply. MSC-Exos can deliver miR-532-5p, which targets RASSF5, and eventually inhibit TNF-α-induced NPC apoptosis. By delivering miR-142-3p to target MLK3 in NPCs, MSC-Exos inhibit the activation of the MAPK pathway and alleviate IL-1*β*-induced apoptosis and inflammation in NPCs. MSC-Exos can transfer miR-21, which directly targets PTEN; the PTEN silencing actives PI3K/Akt pathway and suppresses activation of Bad, Bax, and caspase-3 and inhibits TNF-α-induced NPC apoptosis. **(B)** Inhibiting ECM degradation. BMSC-Exos suppress the levels of MMP-1, MMP-3, MMP-13 in degenerative NPCs to inhibit ECM decomposition metabolism and evaluate expression levels of aggrecan, collagen II, SOX9, further inhibiting the ECM degradation. **(C)** Inhibiting inflammation response. MSC-Exos can decrease inflammatory factor expression including IL-1*α*, IL-1*β*, IL-6, IL-17, NF-κB-p65 and TNF-*α* in NPCs. MSC-Exos can inactivate the NLRP3 and inhibit the expression of NT-GSDMD, IL-18 and IL-1*β* proteins in degenerative NPCs, therefore inhibiting the NLRP3-mediated inflammatory pyroptosis. **(D)** Inhibiting oxidative stress. MSC-Exos can reduce the ROS and MDA level and inhibit oxidative stress-induced NPC apoptosis. MSC-Exos can activate Akt/ERK pathway to decrease CHOP protein expression, therefore inhibiting the cleavage of caspase-3, caspase-12 to treat AGEs-related ER stress-induced IDD. **(E)** Promoting chondrogenic differentiation. MSC-Exo treatment can induce chondrogenesis in degenerative NPCs earlier by increased the aggrecan, collagen II and SOX9 expression. **(F)** Protective effect on endplate chondrocytes. MSC-Exos containing miR-31-5p could negatively regulate ER stress by targeting ATF6, and further reducing caspase-3, caspase-7 and caspase-9 expression to inhibit apoptosis and calcification of endplate chondrocytes. **(G)** Protective effect on annulus fibrosus of IDD. BMSC-Exos can suppress PI3K/Akt/mTOR signaling pathway-mediated autophagy and inhibit the IL-1β-induced inflammation and apoptosis in annulus fibrosus cells.

Low back pain is a global health hazard (Manchikanti et al., 2014). Disc herniation and spinal stenosis caused by IDD are the main causes of low back pain, with a high incidence amongst the elderly (Croft et al., 2021). The pathogenesis of IDD is characterized by a decreased number of nucleus pulposus cells (NPCs), extracellular matrix (ECM) decomposition, annulus fibrosus aging, and cartilage endplate calcification (Grunhagen et al., 2011; Yang et al., 2020b). The nucleus pulposus is located at the center of the intervertebral disc and is composed of the proteoglycan elastin and ECM, which are important components responsible for pressure bearing in the intervertebral disc [2]. In IDD, the nucleus pulposus is the first to degenerate, mostly following an abnormal amount of stress on the vertebrae, aging, nutrition and other factors (Urban and Roberts, 2003). The exosomes derived from MSCs contain a variety of regulatory factors that inhibit the development of IDD. Studies have shown that MSC-Exos could prevent IDD by inhibiting apoptosis and promoting the proliferation of NPCs, inhibiting ECM degradation, alleviating inflammatory response and oxidative stress, promoting chondrogenic differentiation, and protecting endplate chondrocytes and annulus fibrosus. Therefore, MSC-Exos may be a promising option to delay or even reverse IDD (Hu et al., 2020; Krut et al., 2021). The related mechanisms are described in detail below and displayed in Figure 2.

### 2.2 Potential Mechanisms of Action of MSC-Exos in the Treatment of Intervertebral Disc Degeneration

#### 2.2.1 Inhibiting Nucleus Pulposus Cell Apoptosis and Promoting Nucleus Pulposus Cell Proliferation

The main pathophysiologic mechanism of IDD involves a decline in the number of NPCs and ECM degradation, with multiple reactions such as inflammation and oxidative stress also being involved in this process. Therefore, inhibition of nucleus pulposus cell apoptosis and promotion of cell proliferation is the focus of IDD treatment. Exosomes derived from bone marrow mesenchymal stem cell (BMSC-Exos) have been reported to increase the proliferative ability of NPCs along with increasing the concentration of MSC-Exos (Li et al., 2020a; Hu et al., 2021). For exosomes derived from adipose-derived mesenchymal stem cells (ADMSC-Exos), the proliferation and migration rate of human NPCs were elevated through the ADMSC-Exo treatment (Zhang et al., 2021c). Furthermore, BMSC-Exos were able to prevent and mitigate NPC apoptosis induced by acidic pH by repressing caspase-3 expression and attenuating caspase-3 cleavage. When NPCs are placed in the pathological state of low blood supply, BMSC-Exos could increase miR-155 expression in NPCs, thereby downregulating Bach1 expression and upregulating heme oxygenase-1(HO-1) expression, activating autophagy in NPCs, inhibiting the level of apoptosis, thereby inhibiting IDD (Shi et al., 2021). Besides, BMSC-Exos could reduce the apoptosis rate of NPCs induced by tumor necrosis factor-*α* (TNF-*α*), while the miR-532-5p level was decreased in apoptotic NPCs. RASSF5 was demonstrated as a target of miR-532-5p; BMSC-Exos may inhibit apoptosis by targeting RASSF5 to deliver miR-532-5p to inhibit NPC apoptosis (Zhu et al., 2020a). BMSC-Exos also could alleviate interleukin-1*β* (IL-1*β*)-induced apoptosis and inflammation in NPCs, which may be mediated by delivering miR-142-3p to target mixed-lineage kinase-3 (MLK3) in NPCs and further inhibiting mitogen-activated protein kinase (MAPK) signaling (Zhu et al., 2020b). Using MSC-Exos enriched in miR-21 to transfer miR-21 to TNF-α-induced NPCs, the apoptosis level in the NPCs could be downregulated (Cheng et al., 2018). In this process, miR-21 directly targets phosphatase and tensin homolog (PTEN), which is negatively regulated by miR-21. The PTEN silencing actives phosphoinositide 3-kinases (PI3K)/ protein kinase B (Akt) pathway then decreases the activation level of downstream factors of Bad, Bax and caspase-3, and finally inhibit TNF-α-induced apoptosis.

#### 2.2.2 Inhibiting ECM Degradation

Disc height is reduced due to the loss of matrix, which is mainly caused by matrix metalloproteinases (MMPs), which can hydrolyse ECM components such as proteoglycan collagen, thereby accelerating the pathological process of IDD (Kozaci et al., 2006). BMSC-Exo treatment can promote the expression levels of anabolic/matrix protective genes including aggrecan, collagen II, SRY-box transcription factor 9 (SOX9); suppress the levels of matrix-degrading genes such as MMP-1, MMP-3, MMP-13 in degenerative NPCs (Lu et al., 2017; Li et al., 2020a). Moreover, studies have shown that lactic acid accumulation can reduce the pH value in IDD (Malandrino et al., 2014). Acidic pH adversely affects the proliferation of NPCs, and destroys the metabolic balance of the ECM, which limits the therapeutic potential of MSCs and is a negative factor affecting intervertebral disc repair (Huang et al., 2013). Moreover, ADMSC-Exos have also been found to suppress the MMP-13 expression, inhibit ECM decomposition in degenerative NPCs, and increase collagen II expression to promote ECM formation (Xing et al., 2021).

#### 2.2.3 Inhibiting Inflammation Response

Previous researches have confirmed inflammation and related signaling pathways as important factors in the onset and progression of IDD, an obvious etiologic factor of low back pain (Lyu et al., 2021). Studies have demonstrated that the secretion of inflammatory factors such as IL-1*α*, IL-1*β*, IL-6, IL-17, nuclear factor-κB p65 (NF-κB p65), TNF-*α* was increased in the NPCs from degenerative discs, and found that ADMSC-Exos could decrease the inflammation level (Zhang et al., 2021c). Additionally, ADMSC-Exos could inactivate the NLRP3 inflammasome, inhibit the expression of N-terminal gasdermin D (NT-GSDMD) and IL-1*β* proteins in degenerative NPCs, thereby more significantly reducing the inflammatory response (Xing et al., 2021). Besides, it was also shown that MSC-Exos significantly decrease NLRP3 expression and reduce caspase activation, hence downregulating the expression levels of downstream cytokines IL-18 and IL-1*β*, inhibiting NLRP3-mediated inflammatory pyroptosis in the degenerative NPCs (Zhang et al., 2020a). And the establishment of an ECM hydrogel system could sustainably release ADMSC-Exos, allowing exosomes to remain in the degenerative disc for up to 28 days to exert a more anti-inflammatory effect.

#### 2.2.4 Inhibiting Oxidative Stress

Reactive oxygen species (ROS) is a crucial factor for intervertebral disc signal transduction, and the excessive production of ROS can accelerate IDD (Suzuki et al., 2015; Feng et al., 2017). Finding therapeutic targets to reduce excessive ROS is a valuable research orientation, which could work mainly by inhibiting oxidative stress. Hu et al. (Hu et al., 2021) found that BMSC-Exos could reduce ROS and malondialdehyde (MDA) level and inhibit oxidative stress-induced NPC apoptosis. Besides, suppressing the Akt/extracellular signal-regulated kinase (ERK) pathways was demonstrated to aggravate endoplasmic reticulum (ER) stress-induced apoptosis (Xu et al., 2017). MSC-Exos could protect the NPCs against advanced glycation end products (AGEs)-related ER apoptosis by activating Akt/ERK signaling, which could reduce C/EBP homologous protein (CHOP) expression, and attenuate the cleavage of caspase-3, caspase-12 (Liao et al., 2019). Therefore, BMSC-Exos may be the effective therapeutic method to treat AGEs-related ER stress-induced IDD.

#### 2.2.5 Promoting Chondrogenic Differentiation

The reduction of chondrogenic NPCs and the lower expression of chondrogenic genes were the critical manifestations of IDD (Maldonado and Oegema, 1992; Choi et al., 2015). An important function of chondrocyte-like NPCs was to produce ECM, and more chondrocyte-like NPCs can produce more ECM (Adams and Roughley, 2006). SOX9 is one of the early markers of chondrogenesis for NPCs (Chimal-Monroy et al., 2003). It was reported that MSC-Exos could promote SOX9 expression in NPCs from degenerative NP tissue more quickly, meaning that MSC-Exo treatment can induce earlier chondrogenesis in degenerative NPCs (Hingert et al., 2020). A study (Zhang et al., 2021c) reported that after treatment of NPCs with ADMSC-Exos for 7, 14 and 21 days, the levels of chondrocytic genes (collagen II, aggrecan and SOX9) were significantly increased, suggesting that ADMSC-Exos had restored the chondrogenic differentiation properties of degenerative NPCs.

#### 2.2.6 Protective Effect on Endplate Chondrocytes

Degenerative changes of the cartilage endplate can hinder nutrient transfer to the intervertebral disc and aggravate IDD (Zhu et al., 2016; Wong et al., 2019). It was reported that MSC-Exos containing miR-31-5p could negatively regulate activating endoplasmic reticulum (ER) stress by targeting transcription factor 6 (ATF6), and further inhibit expression of caspase-3, caspase-7, and caspase-9, thereby inhibiting tert-butyl hydroperoxide-induced apoptosis and calcification in endplate chondrocytes (Xie et al., 2020). By injecting MSC-Exos to the sub-endplate of the IDD model in rat tails, the MSC-Exos displays an inhibiting effect on IDD. Conversely, the protective effects were reduced when the miR-31-5p levels were downregulated in MSC-Exos.

#### 2.2.7 Protective Effect on Annulus Fibrosus of Intervertebral Disc Degeneration

Due to complicated biomechanics, both the number of cells in the annulus fibrosus and nucleus pulposus are found to be considerably decreased during the IDD (Vergroesen et al., 2015). Gene analyses have delineated that autophagy-related gene expression is significantly increased in degenerative annulus fibrosus tissues. The number of autophagic vesicles and autophagosomes was enhanced, suggesting that autophagy may play an essential role in the pathogenesis of IDD (Gruber et al., 2015). Research has equally revealed that, BMSC-Exos could inhibit IL-1*β*-induced inflammation and apoptosis and promote the proliferation of annulus fibrosus cells, thus exerting a protective effect on the annulus fibrosus, and this may be by suppressing PI3K/Akt/mTOR signaling pathway-mediated autophagy (Li et al., 2020b).

## 3 Mesenchymal Stem CELL-DERIVED Exosomes and Bone Repair and Regeneration

### 3.1 Relationship Between MSC-Exos and Bone Repair and Regeneration

**FIGURE 3:**
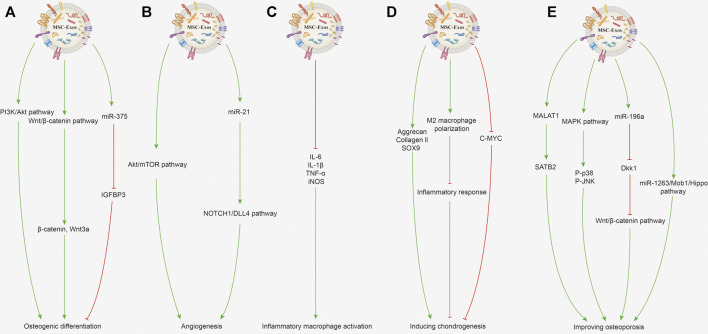
Potential mechanisms of MSC-Exos for promoting bone repair and regeneration. **(A)** Promoting osteogenic differentiation. MSC-Exos can activate the PI3K/Akt signaling pathway to promote osteogenic differentiation and proliferation of BMSCs. The Wnt/*β*-catenin signaling pathway activated by MSC-Exos with increased expression of β-catenin and Wnt3a can promote osteoblast proliferation and differentiation. Exosomes enriched with miR-375 inhibit the IGFBP3 expression to promote osteogenic differentiation of hBMSCs. **(B)** Promoting angiogenesis. BMSC-Exos activates the Akt/mTOR pathway to stimulate angiogenesis and further promotes bone regeneration. Exosomal miR-21 can upregulate the NOTCH1/DLL4 pathway and promote angiogenesis. **(C)** Immunoregulation. MSC-Exos can decrease the gene expression of IL-1*β*, IL-6, TNF-*α*, and iNOS in the inflammatory macrophages. **(D)** Inducing chondrogenesis. MSC-Exos increase the protein and mRNA expression of collagen II and SOX9 to improve cartilage regeneration. MSC-Exos can promote the macrophage’s polarization toward the M2 phenotype and further inhibit the inflammatory response to benefit chondrogenesis. BMSC-Exo treatment can decrease the c-MYC expression level, which indicates the chondrocyte’s maturation. **(E)** Improving osteoporosis. MALAT1 contained in BMC-Exos promotes alkaline phosphatase activity of osteoblasts and mineralizes nodules by increasing SATB2 expression. Activating MAPK signaling and increasing the P-p38 and P-JNK expression by MSC-Exos can promote osteoblast differentiation. BMSC-Exos enriched with miR-196a can inhibit Dkk1 expression to activate the Wnt/*β*-catenin pathway, thereby improving osteoporosis. Exosomes derived from human umbilical cord MSCs can inhibit BMSC apoptosis through the miR-1263/Mob1/Hippo signaling pathway to improve osteoporosis.

Various bone defects caused by trauma, tumor, infection, congenital deformity and osteoporosis, seriously reduce the life quality of patients and are commonly seen in the clinic (Benjamin, 2010). A small quantity of bone defects or injuries can usually repair themselves, but large and complex bone defects usually need to be filled with artificial or autologous bone, but issues such as bone insufficiency and immune rejection are still encountered. Additionally, osteoarthritis is a common disease of progressive destruction of articular cartilage, accompanied by increased pain, currently lacking effective drugs targeting cartilage repair and regeneration (Li et al., 2013). MSC-Exos are effective at promoting bone repair and regeneration independently, and play an immunomodulatory role by binding with receptors to promote osteogenesis (Zhang et al., 2020b; Fan et al., 2021). Current research suggests that MSC-Exos can promote osteogenic differentiation and angiogenesis, regulate immune function, induce chondrogenesis and improve osteoporosis (Lu et al., 2019; Yang et al., 2021). The related mechanisms are described in detail in the following contents and shown in Figure 3.

### 3.2 Potential Mechanisms of MSC-Derived Exosomes for Promoting Bone Repair and Regeneration

#### 3.2.1 Promoting Osteogenic Differentiation

MSCs have outstanding osteogenic differentiation capacity, and they have been widely used in promoting bone repair and regeneration (Pittenger et al., 1999; Jiang et al., 2002). The miRNAs, growth factors, cytokines contained in MSC-Exos could promote the osteogenic differentiation abilities of MSCs (Wang et al., 2015). Studies have shown that MSC-Exos can profoundly improve the osteogenic differentiation and proliferation of BMSCs, and reinforce the osteogenic response of BMSCs by activating the PI3K/Akt signaling pathway (Zhang et al., 2016). Besides, the exosomes derived from younger BMSCs depicted a stronger ability to promote osteogenic differentiation. It was reported that younger BMSC-Exos (2 weeks) could enhance the proliferation and osteogenic differentiation of older BMSCs (15 months) (Jia et al., 2020). The *in vivo* experiments also verified that bone regeneration was significantly accelerated in rats treated with MSC-Exos. Moreover, activation of the Wnt/*β*-catenin signaling pathway can stimulate osteoblast proliferation and differentiation, and promote bone fracture repair and regeneration (Gaur et al., 2005). The human umbilical cord MSC-Exos treatment could promote the expression levels of *β*-catenin and Wnt3a protein in the Wnt signaling pathway in fracture site cells, indicating that MSC-Exos probably promotes osteoblast proliferation and differentiation as well as bone fracture repair through the Wnt signaling pathway (Zhou et al., 2019). Furthermore, exosomes enriched with miR-375 could promote the osteogenic differentiation of BMSCs by inhibiting insulin-like growth factor binding protein 3 (IGFBP3) expression as a negative regulator of osteogenic differentiation (Chen et al., 2019).

#### 3.2.2 Promoting Angiogenesis

Angiogenesis is a prerequisite for bone regeneration and provides the necessary growth factors and nutrients for the repair of bone injuries and defects (Yu et al., 2009). Besides, new blood vessels serve as a route for transferring the inflammatory cells, and the precursor cells of cartilage and bone, allowing them to reach the site of bone injury. Angiogenesis is regulated by various growth factors, such as various miRNAs, vascular endothelial growth factors (Hankenson et al., 2011). BMSC-Exos stimulates angiogenesis by activating the Akt/mammalian target of rapamycin (mTOR) pathway, which further promotes bone regeneration (Liang et al., 2019). Besides, MSC-Exos can enhance the proliferation, migration, and angiogenic differentiation of endothelial progenitor cells, further driving the process of angiogenesis (Zhang et al., 2021b). Mechanistic studies revealed that exosomal miR-21 promote angiogenesis by upregulating the NOTCH1/DLL4 pathway (Zhang et al., 2021b). It was also found that miR-214-3p was significantly increased in the BMSC-Exos of bone-losing mice. Moreover, knee loading was found to promote angiogenesis and bone regeneration by enhancing the formation of type H vessels and downregulating miR-214-3p levels in BMSC-Exos (Wang et al., 2021a).

#### 3.2.3 Immunoregulation

Bone regeneration and healing is a complicated process, and the levels of cytokines produced in bone injury are first elevated and then gradually decline (Marsell and Einhorn, 2011; Einhorn and Gerstenfeld, 2015). However, continuous or abnormal activation of immune cells or secretion of proinflammatory molecules is detrimental to bone regeneration (Gibon et al., 2017). Macrophages as immune cells play a crucial role in bone regeneration, secreting inflammatory and chemotactic mediators, and initiating the recruitment of MSCs (Loi et al., 2016). MSC-Exos possess a sustained inflammation-regulatory ability, which could decrease the gene expression of IL-1*β*, IL-6, TNF-*α*, and suppress the expression of an M1 phenotypic marker (iNOS) mRNA in the inflammatory macrophages (Wei et al., 2019; Zhang et al., 2020b). The scanning electronic microscopy results depicted that the morphology of macrophages was significantly elongated after treatment with BMSC-Exos.

#### 3.2.4 Inducing Chondrogenesis

Cartilage damage and defect regeneration remain challenges due to its limited healing capacity. (Sun et al., 2010; Chen et al., 2018). Osteoarthritis is one of the most common joint diseases associated with progressive damage and loss of articular cartilage, thus exploring drugs that promote cartilage regeneration could be promising for the treatment of osteoarthritis (Hunter and Bierma-Zeinstra, 2019). MSC-Exos could increase chondrocyte proliferation and improve cartilage regeneration by increasing the protein translation and mRNA expression of hyaline cartilage-specific genes aggrecan, collagen II, and SOX9 (Li et al., 2021; Liao et al., 2021). Similarly, MSC-Exos could promote macrophage polarization toward the M2 phenotype and further inhibit the inflammatory response, creating favorable conditions for osteochondral regeneration (Jiang et al., 2021). In the process of cartilage formation treated with BMSC-Exos, the expression level of c-MYC was reduced, indicating that the exosomes could promote cartilage maturation (Iwamoto et al., 1993).

#### 3.2.5 Improving Osteoporosis

Osteoporosis is caused by complex metabolic factors and is characterized by an obvious decline in bone mineral density and bone microstructure damage (Saito and Marumo, 2010; Hamann et al., 2012). The disease is related to an imbalance between the number and function of osteoblasts and osteoclasts. Moreover, angiogenesis, inflammation, oxidative stress and miRNAs have been involved in the process of osteoporosis (Li et al., 2018; Lu et al., 2021). It was reported that MSC-Exos could promote osteogenesis of BMSCs and promote the proliferation of osteoblasts to alleviate osteoporosis (Qi et al., 2016; Zhao et al., 2018). It was demonstrated that BMSC-exosomal metastasis-associated lung adenocarcinoma transcript 1 (MALAT1) promoted osteoblast activity in osteoporotic mice by the miR-34c/SATB2 signaling pathway (Yang et al., 2019). MALAT1 contained in exosomes derived from BMSCs promoted alkaline phosphatase activity of osteoblasts and mineralized nodules by increasing the expression level of SATB2 (Yang et al., 2019). Previous researches have demonstrated that activating MAPK signaling plays a crucial role in inducing osteoblasts differentiation to reduce and prevent osteoporosis, which may be mediated by increasing the expression levels of P-p38 and P-Jun N-terminal kinase (P-JNK) (Gallea et al., 2001; Zhao et al., 2018). Besides, MSC-Exos could suppress the activation of the NLRP3 inflammasome, inhibit the IL-1β and IL-18 secretion, and alleviate the inflammatory response to improve osteoporosis (Zhang et al., 2021a). Moreover, BMSC-Exos enriched with miR-196a could promote osteogenic differentiation (Peng et al., 2021). Mechanistic studies showed that miR-196a delivered by BMSC-Exos plays an essential role in enhancing osteoblastic differentiation by inhibiting Dkk1 expression to activate the Wnt/*β*-catenin pathway. Exosomes from human umbilical cord MSCs are also able to inhibit BMSC apoptosis and improve the degree of osteoporosis in rats, which was mediated *via* the miR-1263/Mob1/Hippo signaling pathway (Yang et al., 2020a).

## 4 Conclusion

Currently, exosomes are widely viewed as effective therapeutic components derived from MSCs, and the secretion of exosomes is an important way for MSCs to promote the repair of surrounding tissue injuries. There are ongoing researches on the benefits of therapy with MSC-Exos for IDD, as well as bone defects and injuries. The core underlying pathophysiologic mechanism of IDD are abnormalities and a reduced number of NPCs. The functional substance in MSC-Exos can regulate the cell metabolism and function by transferring to NPCs, endplate chondrocytes and annulus fibrosus cells, thus inhibiting IDD. Additionally, MSC-Exos also showed great therapeutic potential in terms of repair in bone defects and injuries *via* promoting osteogenic differentiation and angiogenesis and regulating the immune response, and similar results have been illustrated with respect to its therapeutic and preventive effects against cartilage injuries and osteoporosis. Furthermore, the application of novel biomaterials such as hydrogels could prolong the duration of exosomes at the bone injury site and maintain the function and stability of intracapsular proteins and miRNA. In order to enable MSCs to play a better role in repairing tissue injury, studies should continue the exploration of new methods to promote the delivery of bioactive substances in exosomes more efficient and novel biomaterials that can maintain the physiological state of MSC-Exos.
